# Clinical features and prognostic factors in spinal meningioma surgery from a multicenter study

**DOI:** 10.1038/s41598-021-91225-z

**Published:** 2021-06-02

**Authors:** Kazuyoshi Kobayashi, Kei Ando, Tomohiro Matsumoto, Koji Sato, Fumihiko Kato, Tokumi Kanemura, Hisatake Yoshihara, Yoshihito Sakai, Atsuhiko Hirasawa, Hiroaki Nakashima, Shiro Imagama

**Affiliations:** 1 Department of Orthopaedic Surgery, Nagoya University Graduate School of Medicine, 65 Tsurumai-cho, Showa-ku, Nagoya, Aichi 466-8550 Japan; 2 Department of Orthopaedic Surgery, Chubu Rosai Hospital, 1-10-6, Komei-cho, Minami-ku, Nagoya, Aichi 455-8530 Japan; 3 Department of Orthopaedic Surgery, Japanese Red Cross Nagoya Daini Hospital, Myokencho 2-9, Syowa-ku, Nagoya, Aichi 466-8650 Japan; 4 Department of Orthopaedic Surgery, Konan Kousei Hospital, 137, Takaya-cho, Omatsubara, Takaya-cho, Konan, Aichi 483-8704 Japan; 5 Department of Orthopaedic Surgery, Toyohashi Municipal Hospital, 50 Aza Hachiken Nishi, Aotake-cho, Toyohashi, Aichi 441-8570 Japan; 6 Department of Orthopaedic Surgery, National Center for Geriatrics and Gerontology, 7-430 Morioka-cho, Obu, Aichi 474-8511 Japan; 7 Department of Orthopaedic Surgery, Aichi Medical University Hospital, 1-1, Yazakokarimata, Nagakute, Aichi 480-1195 Japan

**Keywords:** Risk factors, Spinal cord diseases, Neurological disorders, Diagnosis, Prognosis

## Abstract

Meningiomas are benign tumors that are treated surgically. Local recurrence is likely if the dura is preserved, and en bloc tumor and dura resection (Simpson grade I) is recommended. In some cases the dura is cauterized and preserved after tumor resection (Simpson grade II). The purpose of this study was performed to analyze clinical features and prognostic factors associated with spinal meningioma, and to identify the most effective surgical treatment. The subjects were 116 patients (22 males, 94 females) with spinal meningioma who underwent surgery at seven NSG centers between 1998 and 2018. Clinical data were collected from the NSG database. Pre- and postoperative neurological status was defined using the modified McCormick scale. The patients had a mean age of 61.2 ± 14.8 years (range 19–91 years) and mean symptom duration of 11.3 ± 14.7 months (range 1–93 months). Complete resection was achieved in 108 cases (94%), including 29 Simpson grade I and 79 Simpson grade II resections. The mean follow-up period was 84.8 ± 52.7 months. At the last follow-up, neurological function had improved in 73 patients (63%), was stable in 34 (29%), and had worsened in 9 (8%). Eight patients had recurrence, and recurrence rates did not differ significantly between Simpson grades I and II in initial surgery. Kaplan–Meier analysis of recurrence-free survival showed that Simpson grade III or IV, male, and dural tail sign were significant factors associated with recurrence (P < 0.05). In conclusion, Simpson I resection is anatomically favorable for spinal meningiomas. Younger male patients with a dural tail and a high-grade tumor require close follow-up. The tumor location and feasibility of surgery can affect the surgical morbidity in Simpson I or II resection. All patients should be carefully monitored for long-term outcomes, and we recommend lifelong surveillance after surgery.

## Introduction

Meningiomas are benign, slow-growing tumors with an uncertain histogenesis, but arachnoidal cells are thought to be the cell of origin. Spinal meningiomas represent 1.2–12% of all meningiomas and 25–45% of all intradural spinal tumors^1–7^. These tumors most frequently occur in women of mainly 50–80 years old, which is largely attributed to expression of hormone receptors^8–10^. Surgery results in improved neurological outcomes and a low recurrence rate, and is the first choice for treatment of spinal meningiomas^11–14^.

The rate of higher grade meningioma among intracranial tumors treated with surgical resection may be increased by selection bias for larger and more symptomatic tumors. A much smaller size threshold is used for indication of surgical resection in the spinal cord because of the narrower anatomy of this region. Also, since spinal meningioma originates from the dura, local recurrence is likely when the dura is preserved. Therefore, en bloc tumor and dura resection is used to prevent recurrence. In particular, there is a general consensus to perform Simpson grade I resection (macroscopically complete removal of a tumor and its visible extensions, with excision of the dural attachment and any abnormal bone) when possible as the best way to avoid disease recurrence^15,16^. Simpson grade II resection (macroscopically complete removal of the tumor and its visible extensions with coagulation of its dural attachment) is also a consensus if the anatomy of the meningioma does not allow Simpson I removal. However, Simpson grade II resection has been found to have a recurrence rate of 1–8%^6,17,18^.

Given the range of recurrence rates in previous reports, there is a need for a study of long-term outcomes to determine the optimum treatment strategy. The purpose of this study was to analyze clinical features and prognostic factors of spinal meningioma treated at several centers to identify the characteristics and appropriate treatment for this tumor.

## Materials and methods

### Patients

Data from the Nagoya Spine Group (NSG) database were used in the study. Between 1998 and 2018, 116 patients with spinal meningioma underwent surgery at seven NSG centers. Age, gender, symptom duration, sagittal and axial location, histological type, preoperative symptoms, dural tail, neurological examination results, resection classified by Simpson grade^15^, and tumor recurrence were obtained for these cases. The tumor location was classified into dorsal, lateral, ventral, and dumbbell types depending on the location of dural attachment to the tumor^16^. Preoperative magnetic resonance imaging (MRI) was routinely performed using T1W, T2W, and T1 with gadolinium (Gd)-enhanced sequences to investigate anatomical details of the lesions.

All patients in the series were examined neurologically preoperatively and postoperatively at final follow-up. Simpson grade was evaluated based on the description of the surgeon and MRI performed during the first postoperative week in selected cases or 3–6 months after surgery. In preoperative MRI, the dural tail sign was defined using Gd MRI, as described by Goldsher et al.^19^. Surgery was performed by surgeons certified by the Japanese Spine Surgery and Related Research (JSSR) society at each facility. The study protocol was approved by the Ethics committee of Nagoya University Hospital. All procedures involving human participants were conducted in accordance with the ethical standards of the institutional research committee and the 1964 Helsinki Declaration and its later amendments or comparable ethical standards. Informed consent was obtained from all subjects or, if subjects are under 18, from a parent and/or legal guardian.

### Neurological assessment

A preoperative neurological grade was assigned using the modified McCormick scale (grade I = normal gait, II = mild gait disturbance not requiring support, III = gait with support, IV = assistance required, and V = wheelchair needed)^20^. McCormick grades I and II were defined as stable gait with no support required for walking, indicating independent gait ability^21–23^.

### Surgical technique

All surgeries were performed using the conventional posterior midline approach and laminectomy beyond the rostral and caudal ends of the tumor to minimize kinking of the spinal cord by laminotomy and to expose the proximal and distal subarachnoid space to allow dissection to begin^14,18,24^. After the dura was opened, an extraarachnoid tumor was identified as the meningioma. For Simpson grade I resection, the tumor origin (dura) was resected after tumor removal. The defect was reconstructed with an artificial dura that covered the defect extradurally. Depending on the facility, lumbar drainage was often performed to prevent postoperative CSF leakage with subcutaneous CSF accumulation in cases in which artificial dura mater was used^25^. For Simpson grade II surgery, the dura was coagulated to the extent of the dural tail sign after complete removal of the tumor with an ultrasonic aspirator. Then, curettage was performed at the tumor origin, which was thoroughly coagulated. In a case in which calcification was found on preoperative CT, meningioma was strongly suspected and an intraoperative pathological examination was not performed. At the discretion of the surgeon, this examination was performed in cases that did not show calcification on preoperative CT.

### Statistical analysis

Results were analyzed by Student *t* test after calculating means and standard deviations. Chi-square and Fisher exact tests were used for analysis of contingency tables. A one-way ANOVA Bonferroni multiple comparison test was used to compare differences between groups. Kaplan–Meier survival analysis was performed to examine the recurrence-free survival period. Data were analyzed using SPSS Statistics 25 (IBM Corp., Chicago, IL, USA), with P < 0.05 considered to be significant.

## Results

### Demographic data

**Figure 1:**
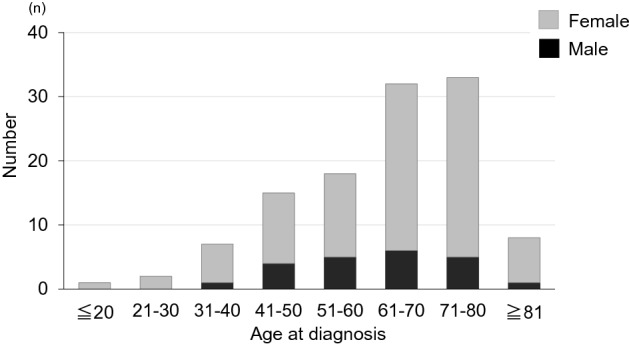
Age distribution of cases by gender.

**Figure 2:**
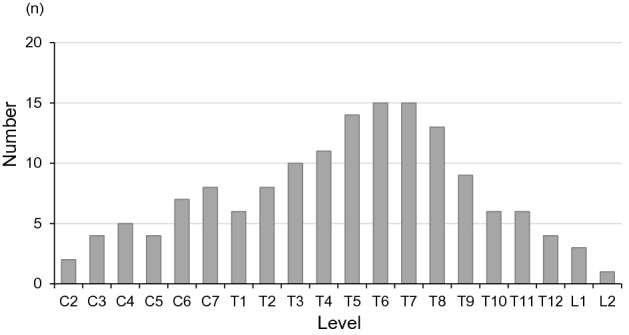
Sagittal tumor location in 116 spinal meningioma (with overlap in some cases).

The 116 patients (22 males, 94 females) ranged in age from 19 to 91 years (mean ± SD 61.2 ± 14.8 years) at the time of surgery (Fig. 1). The sagittal locations of the lesions (Fig. 2) were cervical in 22 cases, thoracic in 90, and lumbar in 4, and the axial location was lateral in 64, ventral in 34, and dorsal in 10 cases. In the WHO classification of histological type, 113 cases were in grade I (61%) (meningothelial: 71, psammomatous: 24, fibrous: 11, transitional: 7), and 3 in grade II (3%) (atypical: 3). A dural tail in Gd MRI was positive in 36 patients (31%). These clinical characteristics are summarized in Table 1.

**Table 1:** Summary of patient characteristics (n = 116).

Characteristics	Value
**Age at diagnosis (years)**	
Mean ± SD	61.2 ± 14.8
Range	19–91
**Gender**	
Male	22 (19%)
Female	94 (81%)
**Symptom duration (months)**	
Mean ± SD	11.3 ± 14.7
Range	1–93
**Sagittal tumor location**	
Cervical	22 (19%)
Thoracic	90 (78%)
Lumbar	4 (3%)
**Axial tumor location**	
Lateral	64 (55%)
Ventral	34 (29%)
Dorsal	15 (13%)
Dumbbell	3 (3%)
**Histological type**	
Meningeothelial	71 (61%)
Psammomatous	24 (21%)
Fibrous	11 (9%)
Transitional	7 (6%)
Atypical	3 (3%)
**WHO classification**	
Grade I	113 (97%)
Grade II	3 (3%)
**Preoperative symptoms**	
Gait disturbance	60 (52%)^†^
Pain	38 (33%)^†^
Weakness	25 (22%)^†^
Sensory deficit	10 (29%)^†^
Urinary disturbance	4 (3%) ^†^
No signs	9 (8%)^†^
Dural tail	36 (31%)

^†^Overlap in some cases.

### Extent of resection and postoperative outcome

**Figure 3:**
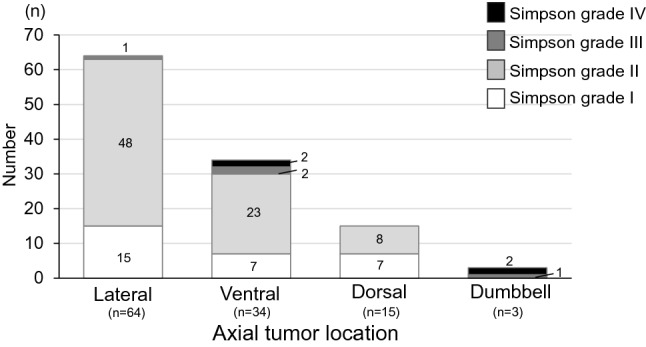
Simpson grade shown by axial tumor location.

Peri- and postoperative data are shown in Table 2. The mean follow-up period was 84.8 ± 52.7 months (range 18–252 months). An intraoperative pathological examination was performed in 20 cases. Complete resection was achieved in 108 cases (94%), including 29 with Simpson grade I and 79 with Simpson grade II resection. Of the other 8 cases, 4 each (4%) underwent Simpson grades III and IV resection. The relationship of axial tumor location and Simpson grade is shown in Fig. 3. Adjuvant radiotherapy was performed in 2 cases in WHO grade II, and the overall rate of cases treated with adjuvant therapy was 2%. These cases underwent external-beam radiotherapy (48 Gy, 54 Gy) after subtotal resection. Chemotherapy was not performed. There were no long-term side effects of radiation therapy. During follow-up, the patients had recurrence, but are alive with disease. There were 3 cases with postoperative CSF leakage in which subcutaneous accumulation (including superficial and deep layers) of CSF continued for more than 3 months, and these cases required reoperation for dural and fascia repair due to headache and nausea. Wound complications due to infection also occurred in these 3 cases.

**Table 2:** Summary of surgical data and postoperative follow-up (n = 116).

Characteristics	Value
Follow-up period (months)	84.8 ± 52.7 (18–252)
**Simpson grade**	
I	29 (25%)
II	79 (68%)
III	4 (3%)
IV	4 (3%)
**Adjuvant therapy**	
Radiotherapy	2 (2%)
**Postoperative complication**	
CSF leakage	3 (3%)
Wound complication	3 (3%)
Recurrence	8 (7%)
**Recurrence-free survival**	
1 y	116/116 (100%)
5 y	67/69 (97%)
10 y	20/26 (78%)
**Postoperative neurological change**	
Improved	73 (63%)
Stable	34 (29%)
Worsened	9 (8%)

**Figure 4:**
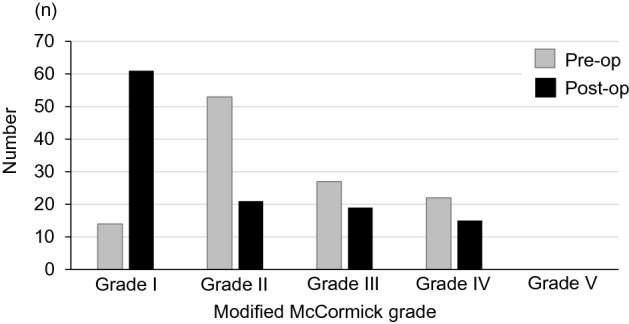
Modified McCormick grade for preoperative and postoperative neurological status.

Results for pre- and postoperative neurological status are shown in Fig. 4. As of the last follow-up, neurological function had improved in 73 patients (63%), was stable in 34 (29%), and had worsened in 9 (8%) (Table 2). The characteristics of cases treated with Simpson II, III or IV resection (that is, not Simpson I) are shown in Table 3.

**Table 3:** Characteristics of cases treated with Simpson II, III or IV resection (n = 87).

Characteristics	Simpson II, III, IV (n = 87)
**Sagittal tumor location**	
Cervical (n = 22)	18 (81%)
Thoracic (n = 90)	69 (77%)
Lumbar (n = 4)	0 (0%)
**Axial tumor location**	
Lateral (n = 64)	49 (77%)
Ventral (n = 34)	27 (79%)
Dorsal (n = 15)	8 (53%)
Dumbbell (n = 3)	3 (100%)
**WHO classification**	
Grade I (n = 113)	84 (74%)
Grade II (n = 3)	3 (100%)

### Tumor recurrence and progression

**Figure 5:**
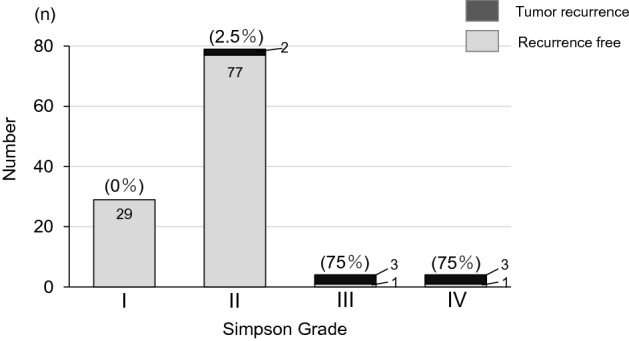
Total number and proportion of tumor recurrence shown by Simpson grade.

Data for 8 patients who underwent reoperation because tumor recurrence or growth exacerbated neurological symptoms are summarized in Table 4. The 8 cases according to histologic type were meningothelial (n = 2, 25%), fibrous (n = 2, 25%), psammomatous (n = 1, 13%), and atypical (n = 3, 38%). The Simpson grades for resection in the 8 reoperations were I (n = 0, 0%), II (n = 2, 2.5%), III (n = 3, 75%); and IV (n = 3, 75%) (Fig. 5). The prognosis was no evidence of disease (NED) in 5 of the 8 patients, and alive with disease (AWD) in the other 3 patients. Two cases in WHO grade II exhibited a high Mib-1 index (8–10%) (Table 4).

**Table 4:** Summary of patients with reoperation due to tumor recurrence.

Case	Age/gender	Simpson grade	Sagittal location	Axial location	Histology	WHO Classification	Time to reoperation (mos)	Ki-67 index (%)	Dural tail	Progn-osis
1	69/M	II	C	Ven	A	II	24	8	−	NED
2	31/F	IV	T	Dum	P	I	63	ND	+	NED
3	41/F	III	T	Dum:	M	I	88	1–2	+	AWD
4	47/F	III	L	Lat	F	I	75	ND	+	NED
5	46/M	III	T	Lat	M	I	115	ND	−	NED
6	49/M	IV	T	Ven	A	II	49	ND	+	AWD
7	58/M	IV	C	Ven	F	I	98	1–2	−	NED
8	57/F	IV	T	Dum	A	II	71	10	+	AWD
Average	49.8						72.9			

*C* cervical, *T* thoracic, *L* lumbar, *Lat* lateral, *Ven* ventral, *Dum* dumbbell, *M* meningeothelial, *P* psammomatous, *F* fibrous, *T* transitional, *A* atypical, *NED* no evidence of disease, *AWD* alive with disease.

**Figure 6:**
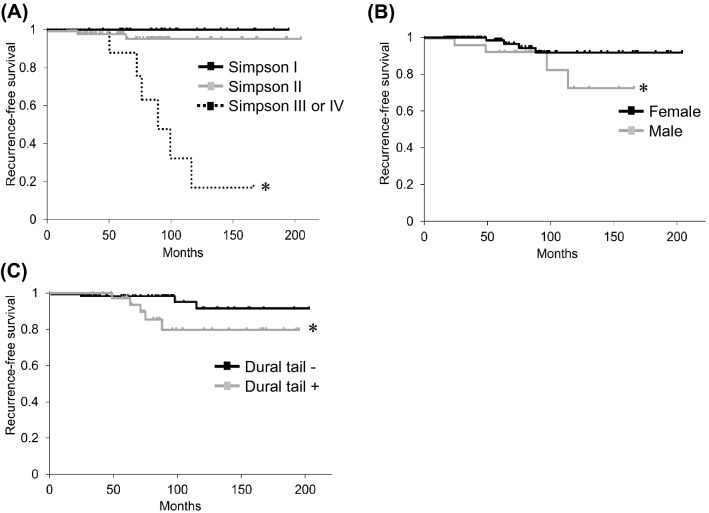
Kaplan–Meier survival curves. Probability of recurrence for (**A**) Simpson grade (I vs. III or IV, P < 0.05; II vs. III or IV, P < 0.05), (**B**) gender (male vs. female; P < 0.05), (**C**) dural tail (dural tail+ vs. dural tail−; P < 0.05). *P < 0.05.

Factors associated with postoperative neurological function and prognostic factors for recurrence are shown in Table 5. A comparison of patients with worsened and improved or stable neurological status indicated that cases with a longer period of preoperative symptoms (P = 0.041) and a higher preoperative McCormick grade (P = 0.024) were more prone to deterioration of neurological function. Comparison of patients with and without recurrence showed that age < 50 years (P = 0.054), male gender (P = 0.018), dural tail (P = 0.046), and Simpson grade (P < 0.01) were associated with recurrence (Table 5). Kaplan–Meier analyses of significant factors for recurrence-free survival (Fig. 6) showed that Simpson grade III or IV, male, and dural tail sign were significantly more likely to recur (P < 0.05) (Fig. 6). A review of the literature^5,6,13,16–18,22,23,26–40^ on the relationship between tumor recurrence and length of follow-up is shown in Table 6.

**Table 5:** Factors associated with postoperative neurological function and prognostic factors for recurrence.

Demographic	Neurological function			Recurrence		
	Worsened (n = 9)	Improved/stable (n = 107)	*P* value	Yes (n = 8)	No (n = 108)	*P* value
**Age**			n.s.			0.054
< 50 years	3 (33%)	34 (32%)		5 (62%)	32 (30%)	
≥ 50 years	6 (67%)	73 (68%)		3 (38%)	76 (70%)	
**Gender**			n.s.			0.018*
Male	4 (44%)	18 (17%)		4 (50%)	18 (17%)	
Female	5 (56%)	91 (83%)		4 (50%)	92 (83%)	
**Sagittal tumor location**			n.s.			n.s.
Cervical	1 (11%)	21 (20%)		2 (25%)	20 (19%)	
Thoracic	7 (77%)	83 (77%)		5 (63%)	85 (79%)	
Lumbar	1 (11%)	3 (3%)		1 (13%)	3 (3%)	
**Axial tumor location**			n.s.			n.s.
Ventral	4 (44%)	29 (27%)		3 (38%)	31 (29%)	
Dorsal	3 (33%)	12 (11%)		0 (0%)	15 (14%)	
Others	2 (22%)	66 (62%)		5 (62%)	62 (57%)	
**Dural tail**			n.s.			0.046*
Yes	4 (44%)	32 (30%)		5 (63%)	31 (29%)	
No	5 (55%)	75 (70%)		3 (37%)	77 (71%)	
**Symptom duration**			0.041*			n.s.
Mean ± SD (months)	29.7 ± 30.1	9.8 ± 8.0		13.0 ± 18.6	11.2 ± 11.7	
**WHO classification**			n.s.			n.s.
Grade I	6 (67%)	107 (100%)		5 (63%)	108 (100%)	
Grade II	3 (33%)	0 (0%)		3 (37%)	0 (0%)	
**Preoperative McCormick**			0.024*			n.s.
I or II	2 (22%)	65 (61%)		3 (37%)	64 (59%)	
III or IV or V	7 (78%)	42 (39%)		5 (63%)	44 (61%)	
**Simpson grade**			n.s.			< 0.01**
I	2 (22%)	27 (25%)		0 (0%)	29 (27%)	
II	5 (56%)	74 (69%)		2 (25%)	77 (71%)	
III or IV	2 (22%)	6 (6%)		6 (75%)	2 (2%)	

Significant difference *P < 0.05, **P < 0.01.

**Table 6:** Literature review of spinal meningioma.

Author, year, [Reference]	Number of cases	Age	Sex (M/F)	Complete resections (%)	Levels C/CT/T/TL/L	Ventral rate %	Recurrence rate %	Mortality	WHO grade I/II/III	OP complications (%)	Mean FU (mos)	Outcome (improved or stable) (%)
Levy et al., 1982^5^	97	53 (range 15–83)	78:19	82	17/0/73/0/7	36	3.1	1.0	97/0/0	5.1	7	100
Solero et al., 1989^6^	174	56 (range 13–82)	31:143	96.5	26/0/144/0/4	14	6	1.0	173/1/0	2.3	168	86
Roux et al., 1996^23^	54	62 (range 8–85)	11:43	92.6	10/43/1	39	3.7	0	54/0/0	9.2	27	98
King et al., 1998^37^	78	62.5 (range 22–91)	12:66	98	11/0/65/0/2	19.2	1.3	1	75/3/0	ND	132	96.1
Klekamp et al., 1999^27^	117	Mean 57 (range 17–86)	24:93	89	32/0/78/0/7	27	15.3	1.7	117/0/0	11.2	156	ND
Gezen et al., 2000^34^	36	Mean 49 (range 20–74)	9:27	97	5/2/20/4/5	19	5.5	3	36/0/0	8.3	108	86.1
Cohen-Gadol et al., 2003^28^	80	Mean 50.8 (range 9–89)	12:68	87.6	24/0/55/0/2	31.7	13.7	0	80/1/0	13.6	82	90
Gottfried et al., 2003^13^	25	60 (range 49–62)	1:4.2	92	4/0/19/0/2	16	4	0	25/0/0	0	23	92
Peker et al., 2005^22^	41	Mean 50 (range 16–73)	9:32	98	7/0/34/0/0	81	ND	0	41/0/0	9.7	23	100
Setzer et al., 2007^26^	80	Mean 61.9 (range 20–91)	22:58	95	17/6/48/6/3	58.8	10	1.2	70/6/4	5	44	93.5
Yoon et al., 2007^18^	38	Mean 52 (range 19–80)	7:31	84.2	6/2/28/1/1	13	15.8	5.3	36/0/2	10.5	73	94.7
Sandalcioglu et al., 2008^32^	131	Mean 69 (range 17–88)	17:114	97	21/7/95/6/2	38	3	0.8	129/2/0	3	60	96.2
Boström et al., 2008^40^	61	Mean 61 (range 28–80)	11:50	98.36	ND	21.3	8.2	0	61/0/0	3.3	84	100
Maiuri et al., 2011^31^	117	Mean 59 (range 18–84)	30:87	94.9	25/0/90/0/2	7.8	3.6	0.8	115/2/0	ND	ND	ND
Postalci et al., 2011^29^	46	Mean 52 (range 17–76)	13:33	82	4/0/39/0/3	15.2	17	0	44/2/0	15	60	91
Nakamura et al., 2012^16^	68	Mean 56 (range 1–82)	12:56	91	14/0/50/0/4	55.9	9.7	0	67/0/1	ND	144	ND
Riad et al., 2013^17^	15	67 (range 28–85)	2:13	100	2/0/11/0/2	46	8	0	15/0	ND	99	100
Iacob, 2014^36^	32	55 (range 34–82)	4:28	100	ND	18.75	6.25	ND	32/0/0	6.25	24	100
Arima et al., 2014^35^	23	Mean 60.3 (range 21–84)	8:15	90	14/0/9/0/0	65.2	ND	0	20/3/0	ND	84	95.7
Maiti et al., 2016^30^	38	Mean 53.5 (range 12–92)	7:31	97.4	10/4/24/0/0	36.8	10.5	0	35/3/0	10.5	51	100
Raco et al., 2017^38^	173	Mean 55.6 (range 16–80)	35:138	98.8	48/0/124/1	50.9	2.3	0	170/2/1	4	50.6	93.1
Santos et al., 2018^33^	51	Mean 57.7 (range 12–92)	11:40	86	15/4/32/0/0	ND	ND	0	48/3/0	11.7	46	96
Voldřich et al., 2020^39^	84	Mean 64 (range 25–86)	16:68	92	15/69/0	ND	8.7	4.7	81/3/0	23	36	98
This study	116	Mean 61.2 (range 19–92)	22:94	93.1	22/0/90/0/4	55.1	6.9	0	113/3/0	5.1	84.8	92

*C* cervical, *CT* cervicothoracic, *T* thoracic, *TL* thoracolumbar, *L* lumbar, *ND* no data.

## Discussion

Spinal meningiomas generally respond favorably to surgical excision and have a low recurrence rate of 3–15.3% after excision^5,6,23^. In our series, in postoperative follow-up of a mean of 84.8 months, the recurrence rate was 6.9% (8/116 cases). The first choice of treatment for spinal meningiomas is complete surgical resection. The recurrence rate associated with subtotal resection is significantly higher than that with total resection^6,24,26,27^, and complete removal of the attached dura (Simpson I) may therefore be the most effective surgical method. In our series, in 29 patients with Simpson grade I resection, there was no tumor recurrence, and only 2 (3%) of 79 patients with Simpson grade II resection developed recurrence. Simpson II resection is also acceptable in spinal meningioma surgery^39^ and results in low recurrence rates^14,27^. Therefore, it is uncertain if Simpson grade I or II resection should be performed. However, if tumor cells can survive coagulation techniques in dura preservation, Simpson II resection might have a risk of leaving residual tumor cells that, although clinically silent, could be a source for recurrence^41^. Yamamuro et al.^41^ and Nakamura et al.^16^ found dural invasion in 19/25 (76%) and 15/43 (35%) cases, respectively. These results suggest that late-developing tumor recurrence after Simpson grade II resection was attributable to the presence of residual tumor cell infiltrates between the inner and outer dural layers. In our series, we found no significant difference between Simpson grade I and grade II cases using Kaplan–Meier survival curves (Fig. 6).

In our series, there was a high rate of WHO grade 1 histology among spinal meningiomas (97%). This could be due to selection bias of larger and more symptomatic tumors for surgical resection, as many small and indolent meningiomas are not resected. However, in the spinal cord, a much smaller size threshold is used to indicate surgical resection, given the narrower anatomy. This may explain the higher than expected rate of WHO grade 1 meningiomas and the high female-to-male ratio in our cohort.

Multiple clinical factors have been associated with increased recurrence rates. Maiti et al. found that males are more likely to have recurrence, despite the lower tumor incidence^30^. In younger patients, Cohen-Gadol et al. showed that extradural extension was associated with a higher recurrence rate^28^. Klekamp and Samii found that arachnoid scarring was significantly associated with increased recurrence^27^. Regarding the tumor location, ventral meningiomas have a higher risk of recurrence than dorsal or lateral meningiomas^16,27,42^. Nakamura et al. showed that 6 of 17 ventral meningiomas had recurrence after Simpson grade II resection^16^, and Postalci et al. reported a recurrence rate of 62% for ventral meningiomas^29^. In our series, the recurrence rate was higher for males and younger patients (< 50 years), but ventral meningiomas did not have significantly greater recurrence. Histopathologically, most spinal meningiomas are in WHO grade I, and Setzer et al. reported recurrence rates of 1.4%, 50%, and 100% for WHO grade I, II, and III lesions, respectively^26^, with the grade being an independent predictor of recurrence.

The three WHO grade II cases in this study had a dumbbell shape tumor with foraminal extension, and waveform deterioration in intraoperative transcranial motor evoked potential (Tc-MEP) spinal cord monitoring during tumor resection that did not recover. Therefore, Simpson grade I resection could not be performed and all three cases ultimately had recurrence (Table 5). Given the high recurrence rate for WHO grade II tumors, complete resection should be attempted when possible.

The postoperative neurological outcome for spinal meningioma is generally favorable, with a clear possibility of recovery even in patients with deep functional preoperative impairment^23,43^. In our series, neurologically independent gait ability (McCormick I and II) at the last follow-up was achieved in 83% of cases (96/116), and among all patients, 92% (107/116) had improved or stable postoperative neurological function. These results are as favorable as those in previous reports. However, large tumors with ventral attachment causing spinal cord signal changes are associated with poor functional outcomes and increased risk of spinal cord traction and surgical damage. In our series, severe preoperative impairment (McCormick grades III, IV and V) was significantly related to neurological deterioration postoperatively. Severe preoperative impairment may reflect plasticity and a vulnerable spinal cord. Complete resection may have led to functional recovery and a better prognosis.

Total resection is possible for most spinal meningiomas^29,36^. Dorsal or dorsolateral lesions are easier to resect, but for ventral lesions sequential debulking and dissection may be helpful. Regarding postoperative complications, the role of resection of the dural attachment is controversial, and contrasting results have been reported. Nakamura et al. found a lower recurrence rate for Simpson grade I resection than for Simpson grade II^16^. In contrast, King et al. reported a low recurrence rate even when dural resection was not performed^37^. Originally, resection of the dural attachment with suturing of a patch graft was advocated^30^. Dura-splitting dissection was also suggested to be useful in cases in which the margin of the tumor is excised in continuity with the inner layer of the dura, and preservation of the outer layer of the dura minimizes the post-resection dural defect, which prevents CSF leakage^44^. However, complete resection of the tumor and associated dura (Simpson I) is limited by postoperative complications, especially for ventral meningiomas. However, in our series, Simpson grade 3 resection was performed in some cases with lateral lesions if waveform deterioration in intraoperative Tc-MEP monitoring occurred without recovery. In such cases, we were forced to interrupt the procedure without achieving total resection.

In our series, Simpson grade I resection was less frequently performed for ventral meningiomas (20%, 13/64). When the tumor was located ventrally, dural resection could not be attempted in most cases. Simpson grade I removal can be technically challenging, because of neurological complications, pseudomeningoceles or CSF leakage. CSF leakage and wound complication are most common, occurring in 0–4% and 0–6% of cases, respectively^30,44^. In our series, reoperation due to CSF leakage was required in three patients, even after Simpson grade I removal. Given these results, Simpson grade I resection should be planned for dorsal lesions, and Simpson grade II resection might be an alternative in a case at high risk for postoperative complications.

Spinal meningiomas are generally isointense to the spinal cord on T1- and T2-weighted images, and show enhancement after administration of contrast agent^26^. A dural tail or linear enhancement of the adjacent dura may appear after Gd administration, similarly to intracranial meningiomas, and its presence often distinguishes meningiomas from other intradural extramedullary lesions^19,45^. Nakamura et al.^16^ also found that preoperative detection of tumor cell invasion between the inner and outer dura mater layers is useful for selecting the operative procedure. Tumor invasion in the dura occurred in 1/3 of spinal meningiomas and in 47% (7/15) of spinal meningiomas with a dural tail sign; therefore, histological analyses of resected dura can clarify the significance of the dural tail sign^16^. In our series, we routinely coagulated the dura to the extent of the dural tail sign in Simpson grade II resection. Our retrospective study lacks histological analysis for the resected dura, but our data show that a dural tail in MRI was significantly more common in recurrence cases. Therefore, the presence of a dural tail should be carefully analyzed in predicting recurrence, and patients with a dural tail on imaging should undergo careful long-term follow-up.

Postoperative mortality in spinal meningioma surgery is usually low, ranging from 0 to 4.7% in series reported later than 1999^6,7,38–40^. Radiotherapy is typically reserved for management of high-grade and recurrent spinal meningioma, and is useful in cases with macroscopic remnants of histological high grade lesions, recurrent meningiomas after subtotal primary excision, or as a therapeutic approach if surgery is unsafe for clinical or anatomic reasons^23^. Reports of chemotherapy for spinal meningiomas are limited, but the outcome seems not to be satisfactory. In our series, two WHO grade II cases received radiotherapy, and both patients are still alive.

This study has several limitations. First, the retrospective design does not offer the advantages of a prospective study. Second, although multiple reports suggest that WHO Grade II meningiomas are prone to recurrence, we had only a few patients in this category, which prevented analysis of many risk factors. In particular, the study included few WHO II meningiomas, and the low statistical power prevented significant findings. Third, a high Ki-67 index is known to correlate with recurrence and histological grade in meningioma^16,46–48^, but in our series the Ki-67 index was not routinely measured. However. the series of 116 patients is relatively large, and we believe that our results provide new information on clinical features and prognostic factors in spinal meningioma surgery. A prospective study with a longer follow-up period is needed to confirm the results.

In conclusion, in our series, there was no significant difference in recurrence rate between Simpson grade I and grade II cases. However, especially in younger patients with long-term follow-up, Simpson I resection is anatomically favorable for spinal meningiomas. MRI can detect a recurrent tumor early, often before clinical symptoms develop. In younger patients, male cases with a dural tail and a histological high grade tumor require closer clinical and MRI follow-up. The tumor location and feasibility of surgery can influence the surgical morbidity in Simpson I or II resection. Even after complete resection of spinal meningioma, patients should be carefully monitored for long term outcomes, and we recommend lifelong surveillance after surgery.
